# Improving the prediction of yeast protein function using weighted protein-protein interactions

**DOI:** 10.1186/1742-4682-8-11

**Published:** 2011-04-27

**Authors:** Khaled S Ahmed, Nahed H Saloma, Yasser M Kadah

**Affiliations:** 1 Department of Bio-electronics, MTI, El-Haddaba Elwosta, Cairo, Egypt; 2 Department of Biomedical photonics, Niles, Giza, (12613), Egypt; 3 Department of Biomedical Engineering, Cairo University, Giza, (12613), Egypt

## Abstract

**Background:**

Bioinformatics can be used to predict protein function, leading to an understanding of cellular activities, and equally-weighted protein-protein interactions (PPI) are normally used to predict such protein functions. The present study provides a weighting strategy for PPI to improve the prediction of protein functions. The weights are dependent on the local and global network topologies and the number of experimental verification methods. The proposed methods were applied to the yeast proteome and integrated with the neighbour counting method to predict the functions of unknown proteins.

**Results:**

A new technique to weight interactions in the yeast proteome is presented. The weights are related to the network topology (local and global) and the number of identified methods, and the results revealed improvement in the sensitivity and specificity of prediction in terms of cellular role and cellular locations. This method (new weights) was compared with a method that utilises interactions with the same weight and it was shown to be superior.

**Conclusions:**

A new method for weighting the interactions in protein-protein interaction networks is presented. Experimental results concerning yeast proteins demonstrated that weighting interactions integrated with the neighbor counting method improved the sensitivity and specificity of prediction in terms of two functional categories: cellular role and cell locations.

## Background

Determining protein functions is an important challenge in the post-genomic era and Automated Function Prediction is currently one of the most active research fields. Previously, researchers have attempted to determine protein functions using the structure of the protein and comparing it with similar proteins. Similarities between the protein and homologues from other organisms have been investigated to predict functions. However, the diversity of homologues meant that these time-consuming methods were inaccurate. Other techniques to predict protein functions including analyzing gene expression patterns [1,2], phylogenetic profiles [3-5], protein sequences [6,7] and protein domains [8,9] have been utilised, but these technologies have high error rates, leading to the use of integrated multi-sources [10,11]. The computational approach was designed to resolve the inaccuracy of protein prediction, using information gained from physical and genetic interaction maps to predict protein functions. Recently, researchers have introduced various techniques to determine the probability of protein function prediction using information extracted from PPI. Results from these trials have been promising, but they do not address effective problems including function correlation [12-14], network topology and strength of interaction.

Network topology represents an interaction between proteins and the mechanism of that interaction. Therefore, much information can be extracted from these networks with regards to the strength of the interaction and its contribution to new function prediction, i.e. weighted contribution. A PPI network can be described as a complex system of proteins linked by interactions, and the computational analysis of PPI networks begins with the representation of the PPI network structure [15,16]. The simplest representation takes the form of a network graph consisting of nodes and edges [17]. Proteins are represented as nodes and two proteins that interact physically are represented as adjacent nodes connected by an edge [18]. On the basis of this graphical representation, various computational approaches including data mining, machine learning and statistical methods can be performed to reveal the PPI networks at different levels.

The computational analysis of PPI networks is challenging and faces major problems. The first problem concerns the unreliability of protein interactions derived from large-scale experiments, which have yielded numerous false positive results (Y2H). Secondly, a protein can have more than one function and could be considered in one or more functional groups, leading to overlapping function clusters. The third problem concerns the fact that proteins with different functions may interact. Therefore, a PPI has connections between proteins in different functional groups, leading to expansion of the topological complexity of the PPI networks. *Neighbour counting *is a method proposed by Schwikowski et al. [19] to infer the functions of an un-annotated protein from the PPI. This method locates the neighbour proteins and predicts their assigned functions and the frequency of these functions; the functions are arranged in descending order according to their frequencies. The first *k *functions are considered and assigned to the un-annotated protein. Some papers used this technique with *k *equalling three. This method makes use of information from the neighbours, but it has several drawbacks: (1) it considers the interactions to be of *equal weights*, which is not logical; (2) it does not consider the nature of the function and whether it is dominant; (3) it does not provide a confidence level for assigning a function to the protein. The problem of confidence levels was addressed in [20], where the authors used chi-square statistics to calculate significance levels on the basis of the probability that various functions are present. The chi-square method provides a deeper analysis than the neighbour counting method, but it is less sensitive and specific.

Deng et al. [21] considered various situations for the presence of a certain function for a protein of interest: (1) number of proteins having this function; (2) number of protein pairs (interacting) having the function; (3) number of protein pairs where one has the function and the other does not; (4) number of protein pairs without this function. A weighted sum of these numbers is calculated according to the random Markov field algorithm, which assigns different weights to interactions and overcomes the above problems by considering the entire interaction network [21]. This method considers the frequency of proteins having the function of interest and the neighbours, with less weight being placed on neighbours that are further away. Therefore, it can be used to calculate the probability that an un-annotated protein has a function of interest, and the results are more accurate than those obtained by using neighbour counting or the chi-square method.

This paper presents a new method for predicting protein function based on estimating a weight for the strength of the interaction between proteins in the PPI. The similarity between protein interactions and the connected routers in a certain autonomous number of networks was explored. Applying the idea of a network linked list of protocols such as OSPF (Open Shortest Path First) can allow information concerning surrounding routers to be obtained, according to the principles of cost and level (hop count) [22,23]. The suggested algorithm was compared with the equal weight interactions method to indicate differences in the accuracy of prediction.

## Results

**Figure 1:**
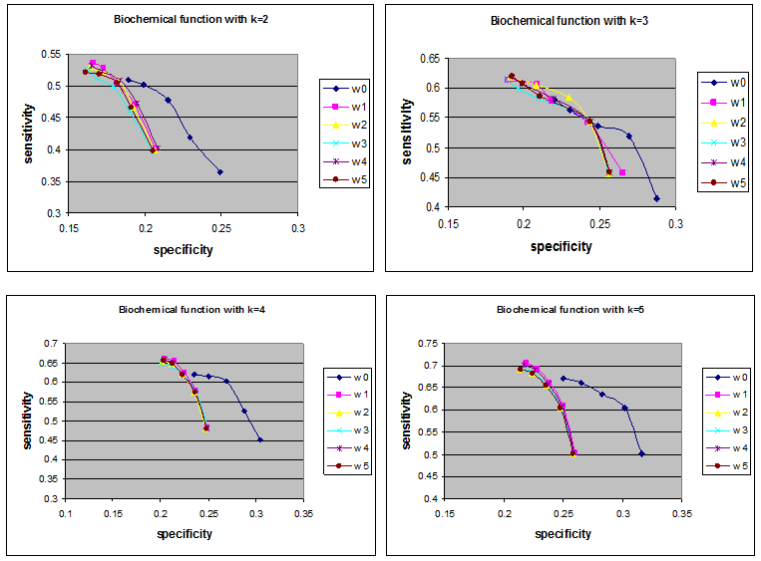
**Biochemical function sensitivity and specificity**. The sensitivity and specificity of the six collected data (un-weighted and five weights) in the biochemical category for up to five interactions (k = 5).

**Figure 2:**
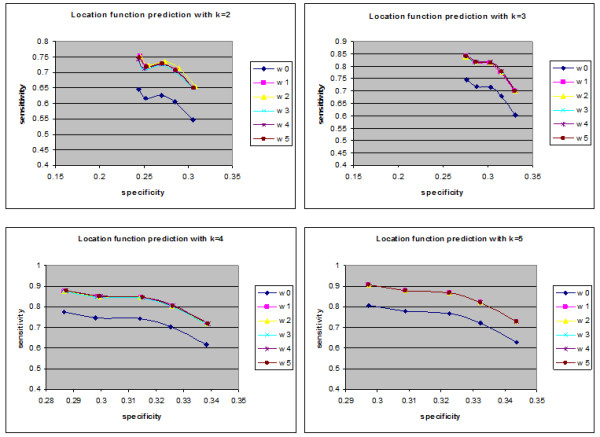
**Cell location function sensitivity and specificity**. The sensitivity and specificity of the six collected data (un-weighted and five weights) in cell location function for up to five interactions (k = 5).

**Figure 3:**
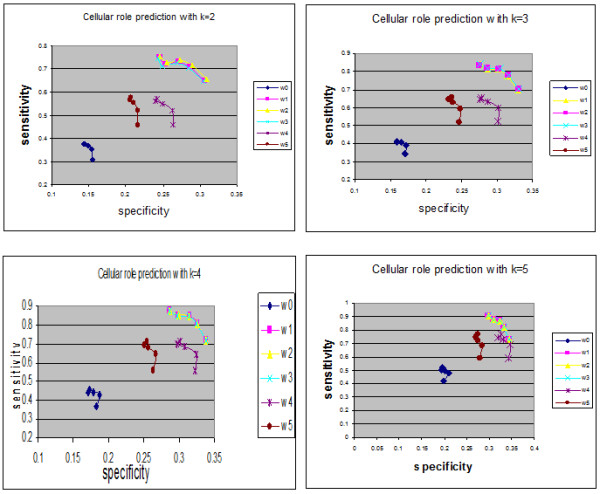
**Cellular role function sensitivity and specificity**. The sensitivity and specificity of the six collected data (un-weighted and five weights) in cellular role category for up to five interactions (k = 5).

The proposed approach was applied to infer the functions of un-annotated proteins in yeast and used weighting interactions rather than free weights (equal interactions). In YPD, proteins are assigned functions based on three criteria: "Biochemical function", "Subcellular location" and "Cellular role". The numbers of annotated and un-annotated proteins, based on the three functional categories, are presented in Table 1. The accuracy of the predictions was measured by the leave-one-out method. For each annotated protein with at least one annotated interaction partner, it was assumed to be un-annotated and functions were predicted using the weighted neighbour counting method. The predicted results were compared with the annotations of the protein. Repeating the leave-one-out experiment for all such proteins allowed the specificity (SP) and sensitivity (SN) to be defined [22]. The corresponding values of *overlapped proteins *for "Biochemical function", "Subcellular location" and "Cellular role" were 1145, 1129 and 1407, respectively. In the first three Figures, the relationship between sensitivity and specificity was implemented for biochemical function, cell location and cellular role, respectively. In terms of the prediction method (neighbour counting method), a fixed number of the highest frequency functions can be compared. In the present study, although one data set is used, k (number of interactions) had a variety of values (from 2 to 5). Figures 1a-d demonstrate the specificity and sensitivity in terms of biochemical function when k equals 2, 3, 4 and 5. In terms of biochemical functions (Figure 1), the sensitivity of a proposed algorithm is higher when specificity values are low. However, for higher specificity the weightless technique (W0) has good sensitivity. Therefore, an established technique is sufficient for predicting biochemical function. As demonstrated in Figures 2 and 3, the sensitivity and specificity for all weights (new suggested techniques W1-W5) were higher than W0 for all values of k. It can be demonstrated that in the cell location function category, W2 (weight relating to IG1) is the best weight to use when the number of interactions for each protein is two. W3 (weights for IG2), W1 (weights for number of experimental method) and W5 (PCA for the basic three weights (W1, W2, W3)) were the best weights when the numbers of interactions for each protein were 3, 4 or 5, respectively. Furthermore, W2 was the best weight for the cellular role function category when the number of interactions was two, and W3 (weights of IG2) were the best weights for the cellular role function category when the numbers of interactions were 3, 4 or 5. There were overlaps between some weights on the indicated curves (overlap curves), but there was a small variation in terms of detecting these weights.

**Table 1:** The numbers of annotated and un-annotated proteins for all proteins, based on three functional categories: Biochemical function, cellular location and subcellular role.

Biochemical function	
Annotated	3353

Un-annotated	3063

**cellular location**	

Annotated	3181

Un-annotated	3235

**Sub-Cellular role**	

Annotated	3894
Un-annotated	2522

## Conclusions

The majority of methods concerning the estimation of protein functions through protein-protein interactions (PPI) use the same weights for all interactions. Such methods do not consider the various situations for each interaction including the number of experimental methods used to identify the interactions, the number of leaves connected to the interaction (whether or not the protein is sticky) and the most common graphs for the studied species within the network. Therefore, this research introduces new weights for protein interactions to enhance protein function prediction. These weights are W1-W5, and W1 depends of the number of experimental methods that identify the interaction. W1 has high confidence (100%) when the number of experimental methods used is more than one. W2 depends on the number of leaves connected to the studied interactions, which indicates whether the protein is sticky or not. The high confidence of W2 is apparent when the IG1 value is less than three (the protein is not sticky). W3 relates to the value of IG2, which indicates the global topology of the network of the studied species; its value is highly confident when IG2 is less than zero. In addition, there are two estimated weights, W4 and W5. W4 is the average of the basic weights (W1, W2 and W3), and W5 is the PCA value for the same weights. Applying the suggested weights to yeast protein functions and integrating these weights with the neighbor counting method led to enhanced results in two function categories: cell location and cellular role. The sensitivity and specificity of every point on the curves of the two function categories were higher than those obtained using the weightless technique (free or equal weights (W0)). W3 was the best weight to use in the cellular role category when the numbers of interactions were 3, 4 or 5. The cell location function category did not have a common weight for all cases but in each case (number of interactions), there was a better weight compared with other methods.

## Methods

**Figure 4:**
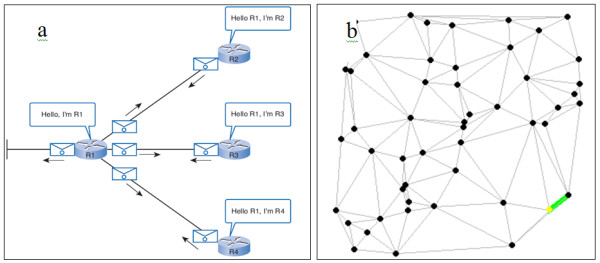
**Connected routers**. Presentation of connected routers in a specific network.

**Figure 5:**
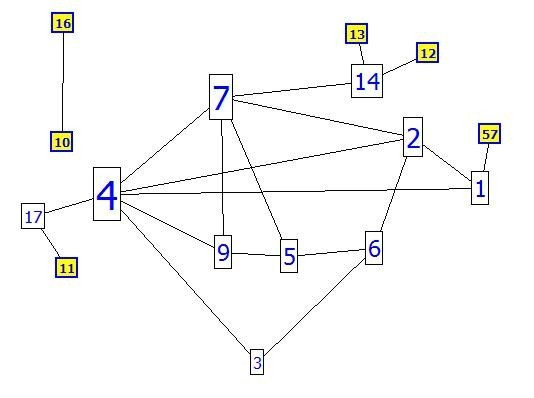
**Connected protein nodes**. Seventeen connected proteins are depicted as a part of the real interacting proteins database, where yellow nodes are leaves (last ones in the path).

This paper introduces a novel algorithm by comparing the proteins in protein-protein interaction networks to the connected routers in the same autonomous number of networking. The protein acts as a router, and the node and edge (interaction between two proteins) act as the connection between two routers (Figures 4 and 5), where routers have up to 100 interactions (29 interactions are the maximum in the yeast proteome). As presented in Figure 4a, a group of routers and their movable messages are indicated, and the connected routers are presented in Figure 4b. In Figure 5, the group of proteins are connected using different experimental methods. The routing system can be introduced by various types of connections (LAN, WAN, Serial) as different experimental methods of interactions in the protein system. Initially, the router will be unaware of neighbour routers on the link. Therefore, the linked state protocol will be applied to the routing system, where a *link *is an interface on a router and the protocols are the control system of all connected routers. The protocol includes information concerning the interface's IP address/mask, the type of network (ethernet (broadcast) or serial point-to-point link), the cost of that link and any neighbour routers on that link. In the protein system, a generic protocol is followed that identifies the protein by name (gene name, locus name, accession name etc...), ID (determined number for each protein), sequence (amino acids in given number and order) and functions (if known). The type of network will be elucidated; interaction between two proteins (protein pair) or dense interactions (cluster), and the weight of the interaction (our contribution). Furthermore, neighbours of the adjacent protein (known interactions in the network) are identified (Table 2). The protein interactions are calculated until the second level. The algorithm is performed following four steps: (1)- determining the level and degree for each adjacent protein, (2)- calculating the weight (cost) for each interaction (interaction with high cost/weight is strong), (3)- integrating these data to predict the function of the un-annotated proteins using the neighbourhood counting method, and (4)- calculating the sensitivity and specificity for the different weights.

**Table 2:** sample of proteins and their interactions

Protein ID	# interactions	p1	p2	p3	p4	p5	p6	p7	P8	p9	p10
32	1	3258	0	0	0	0	0	0	0	0	0
33	23	19	33	33	84	304	333	370	407	568	1065
34	17	56	475	1118	1277	2027	3350	3352	3342	3346	3347
35	0	0	0	0	0	0	0	0	0	0	0
36	5	36	36	2557	3092	4052	0	0	0	0	0
37	0	0	0	0	0	0	0	0	0	0	0
38	0	0	0	0	0	0	0	0	0	0	0
39	0	0	0	0	0	0	0	0	0	0	0
40	1	3802	0	0	0	0	0	0	0	0	0
41	3	1726	3275	386	0	0	0	0	0	0	0
42	0	0	0	0	0	0	0	0	0	0	0
43	0	0	0	0	0	0	0	0	0	0	0
44	0	0	0	0	0	0	0	0	0	0	0
45	0	0	0	0	0	0	0	0	0	0	0
46	1	3708	0	0	0	0	0	0	0	0	0
47	1	4590	0	0	0	0	0	0	0	0	0
48	0	0	0	0	0	0	0	0	0	0	0
49	0	0	0	0	0	0	0	0	0	0	0
50	0	0	0	0	0	0	0	0	0	0	0
51	0	0	0	0	0	0	0	0	0	0	0
52	0	0	0	0	0	0	0	0	0	0	0
53	0	0	0	0	0	0	0	0	0	0	0

### Protein level

**Figure 6:**
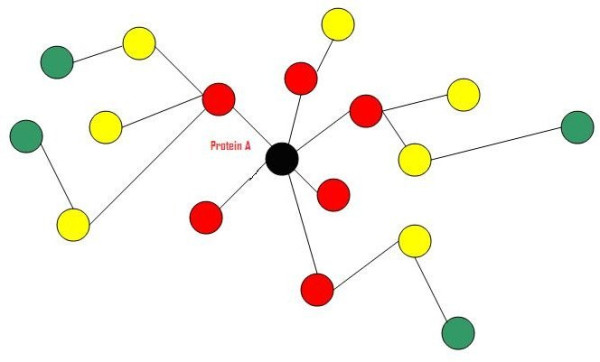
**Protein levels**. Protein A (black) and its surroundings, which were divided into three degrees or levels (red nodes as first level, yellow as second level and green nodes as the third level).

**Figure 7:**
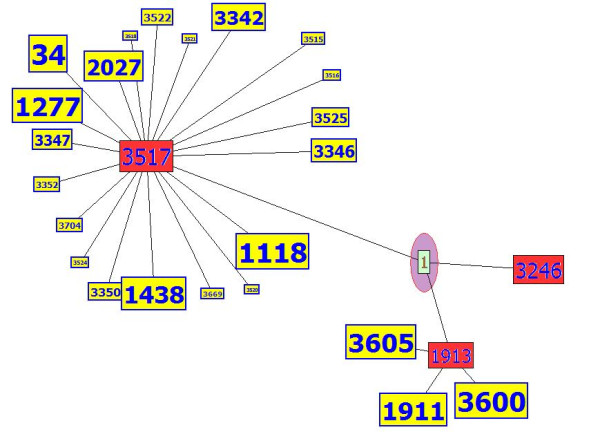
**Saccharomyces cerevisiae network**. A part of the yeast *Saccharomyces cerevisiae *network (MIPS database). The level of the nodes is distributed. The figure has been drawn using the Inter-Viewer program.

There is a difference between the degree and the level of any node. The degree of a node (protein) is defined as the total number of connected nodes or proteins directly surrounding this node (protein A has degree equal to six) as shown in Figure 6; the level of a node is the layer of nodes related to the main one. The directed nodes have a level equal to one, and their neighbours are the second level as presented in Figure 6. The red nodes are the first level of protein A (black), the second level of proteins are the yellow coloured nodes (nodes connected to protein's A neighbours). The last (third) level is the group of proteins coloured green. In router networks, the hop count principle is performed to determine the router level. In this paper, the second level was assumed to be sufficient for extracting the most important information about the function of a protein. The concept of node level was applied to 2559 protein-protein interactions between 6416 proteins collected from the Munich Information center of Protein Sequences (MIPS, http://mips.gsf.de) for the yeast *Saccharomyces cerevisiae *[24]. As demonstrated in Figure 7, proteins with ID numbers 1913, 3246 and 3517 had a level equal to one for the studied protein number 1, and the yellow nodes are second degree.

### PPI weight calculation

**Figure 8:**
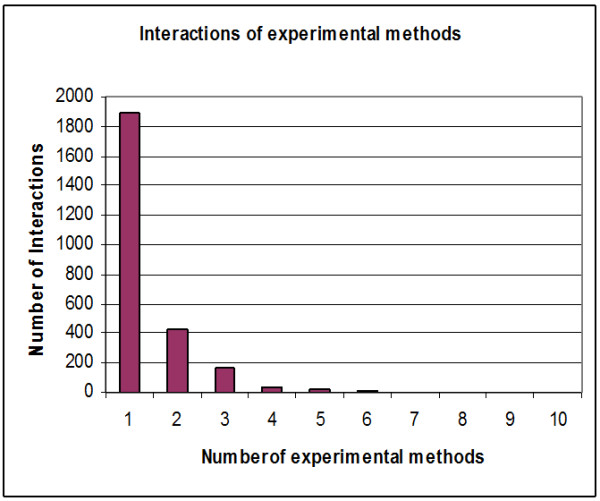
**Interactions/Experimental methods relationships**. Demonstrates the number of interactions (edges) corresponding to the number of experimental methods (~1800 interactions can be identified by one experimental method).

Protein-protein interaction weights are introduced and each interaction has a specific weight. Three basic methods were considered in terms of calculating the weights of all the interactions and overcoming problems affecting the interaction network. The first method concerns the number of experimental methods. Protein-protein interactions are identified by high-throughput experimental methods such as Y2H [25-29], mass spectrometry of co-immunoprecipitated protein complexes (Co-IP) [30,31], gene co-expression, TAP purification cross link, co-purification and biochemical methods. Challenging technical problems arise using the first two methods, which lead to spurious interactions due to self activation in Y2H and abundant contaminants with CO-IP. These problems lead to false positive interactions [32]. Therefore, a quantitative method for evaluating the pathway through proteomics data is required. A number of experimental and computational approaches have been implemented for large-scale mapping of PPIs to realize the potential of protein networks for systems analysis. One method utilizes multiple independent sets of training positives to reduce the potential bias of using a single training set; this method uses association with publishing identifiers or foundation in two or more species, otherwise PPIs must have an expression correlation more than 0.6 [33]. Another technique also obtains conserved patterns of protein interactions in multiple species [34]. There are several methods for determining the reliability of interactions [35-38]. In this paper, the reliability or confidence is introduced by counting the number of experimental methods for each interaction; some interactions have been identified using many experimental methods and others identified by just one. In yeast proteins, approximately ten experimental methods can be used to identify protein-protein interactions (Edge between Protein (YBR0904) and Protein (YDR356W) can be identified by ten experimental methods where protein (AAC1) and protein (YHR005C-A) can be identified by one method). As demonstrated in Figure 8, approximately 750 interactions from 2559 proteins have been identified by more than one experimental method. More than half of all the interactions have been identified by just one method (~1800 interactions); researchers have high confidence (100%) concerning those interactions identified by more than one method and 50% confidence for the others (one method identification). Table 3 presents the yeast protein interactions, the number of experimental methods used to identify them and the identification value for each one. This method does not depend on clear points on computational algorithms, but reflects the strength of interaction from the laboratory viewpoint. Another approach for estimating the reliability of experimental methods concerns calculating the stability of every method.

**Table 3:** Yeast interaction pairs, number of identification methods and type of experimental method

Protein_1	Protein_2	# Identification Method	Y2H	Cross-link	affinity chromo	precipitation	assay	purification	in Vetro	Others
YKL161C	RLM1	1	1	-	-	-	-	-	-	-
AAC1	YHR005C-A	1		1	-	-	-	-	-	-
AAD14	AAD14	1	1	-	-	-	-	-	-	-
AAD6	YNL201C	1	1	-	-	-	-	-	-	-
ABP1	ACT1	3	1	-	1	-	-	1	-	-
ABP1	RVS167	4	1	-	-	1	-	-	-	2
ABP1	SRV2	3	-	-	-	-	-	-	1	2
YER045C	PSE1	1	1	-	-	-	-	-	-	-
ACC1	DMC1	1	1	-	-	-	-	-	-	-
ACC1	SNP1	1	1	-	-	-	-	-	-	-
ACE2	YNL157W	1	1	-	-	-	-	-	-	-
ACS2	SNP1	1	1	-	-	-	-	-	-	-
ACT1	ACT1	4	1	1	1	-	1	-	-	-
ACT1	AIP1	1	1	-	-	-	-	-	-	-
ACT1	BEM1	2	1	-	-	1	-	-	-	-
ACT1	BNI1	1	1	-	-	-	-	-	-	-

**Figure 9:**
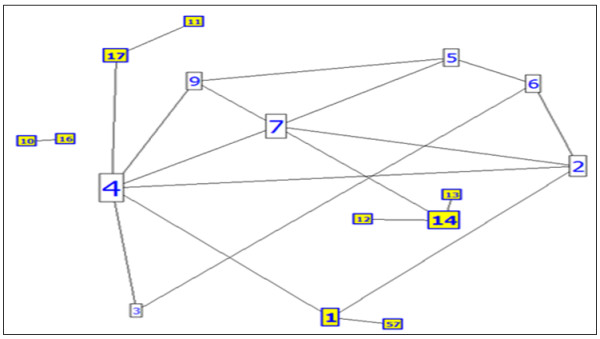
**Interaction Generality 1**. Part of protein IDs and their interactions are presented. The edge between proteins 4 and 17 has an IG1 value of two, where the edge between proteins 7 and 14 has an IG1 value of three.

**Figure 10:**
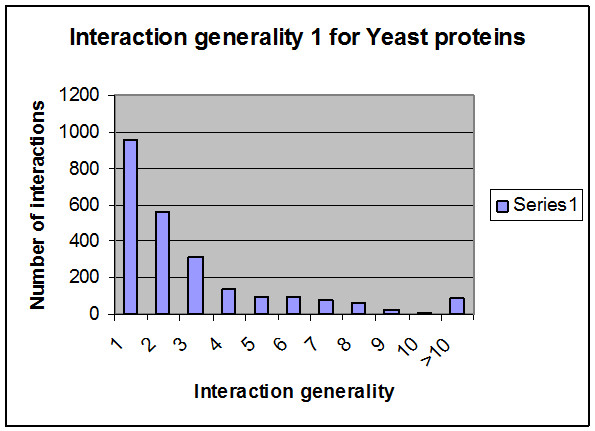
**Interactions/IG1 relationships**. The relationship between the number of interactions and their corresponding IG1values is shown. The last column indicates the number of interactions that have an IG1 of more than 10.

The second method for calculating weights of interactions is the IG1 concept (Interaction Generality 1) [39-41]. A new method for assessing the reliability of protein-protein interactions (local topology) is obtained in biological experiments by calculating the number of proteins involved in a given interaction (number of protein leaves connecting to the two studied proteins incremented by one) as shown in Figure 9. IG1 assumes that complicated interaction networks are likely to be true positives. By implementing the IG1 on the collected data (yeast protein interactions), the range of IG1 was between one and 21 (Figure 10), meaning that some interactions have many leaves. According to the IG1 concept, increasing values leads to false positive interactions. In the suggested algorithm, it is assumed that interactions with an IG1 value less than four (as threshold) have high confidence (100%) and those with more than four have low confidence (Table 4). For example, the interaction between proteins YMR056C and YHRS01C has an IG1 value of three (weight = 100%) when the interaction between proteins YMR056C and YDR167W has an IG1 value of four (weight = 50%). However, the interaction between proteins YDL043C and YMR117C has an IG1 value of 21 (weight = 50%).

**Table 4:** The reliability score of IG1 in protein interactions

PID_1	PID_2	IG1	Reliability score
1	1913	3	1
1	3246	1	1
1	3517	4	0.5
7	7	0	0
19	33	7	0.5
19	2980	1	1
19	3384	1	1
22	2483	2	1
24	785	4	0.5
24	3258	14	0.5
25	5838	2	1
32	3258	13	0.5
33	33	0	0
33	84	7	0.5
33	304	8	0.5
33	333	8	0.5

**Figure 11:**
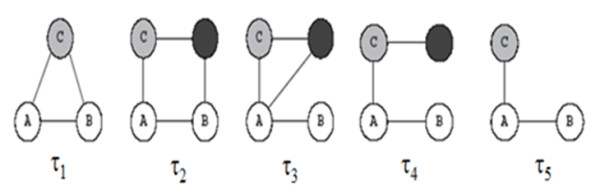
**Interaction generality 2 topologies**. The major five topologies related to the yeast network topology are shown according to interaction generality two.

**Figure 12:**
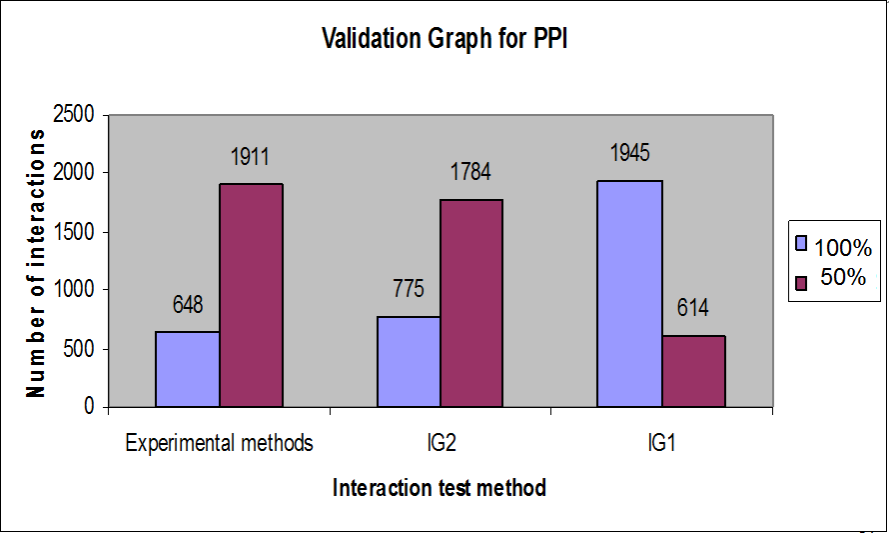
**High confidence edges**. The number of high and low confidence edges for each method used.

The third method for calculating the weight uses the IG2 concept (Interaction Generality 2), [42,43]. This algorithm explores the five major sub-graphs of a network to obtain information concerning the global topology of the network. After collecting the five values for each interaction according to Figure 11, principal component analysis (PCA) has been implemented. The PCA concept for the previous major topologies of yeast protein networks was implemented and IG2 values ranged from -281 up to ~27 (Table 5). By determining the threshold (19) as the margin of reliability, it is assumed that IG2 values less than the threshold are more accurate than those above the threshold. Regarding the three previous methods for calculating weights, high confidence interactions can be collected compared with low confidence interactions (Figure 12). After collecting the weights from the three previous methods (number of experimental methods, IG1 and IG2), new weights strategies can be created using an average of the three values or PCA. Five different weights for each interaction were collected. As indicated in Table 6, interactions between proteins AAC1 and YHR005C-A had a W1 = 0.5, which means that only one method was used to identify it; W2 = 1, therefore it has more than three leaves in IG1 (IG1 < 4), W3 = 0.5 indicating that IG2 was more than 19, W4 is the average of the three weights which equalled 0.66 (1/3 Σ wi, i = 1..3), and W5 (PCA of the three weights with threshold equal zero) was 0.5, indicating that its value is more than zero. This example demonstrates a weak interaction (edge) between protein ID 1 (AAC1) and protein ID 1913 (YHR005C-A). Another example concerning high confidence is shown in the second row and concerns a protein interaction (edge) between ANC1 and SNF5, where the weights are 1, 1, 0.5, 0.83 and 1 for W1, W2, W3, W4 and W5, respectively. Relating to the main three measurements, many weights can be created by applying AND/OR processes. However, each weight can be multiplied by the coefficient relating to the importance of its role in determining the edge; 0.35, - 0.2 and - 0.4 for W1, W2 and W3, respectively. The integration was performed on the five weights explored (W1-W5). The neighbour counting method was applied six times, once for the basic weight (equal weights or traditional method) and once for each of the five estimated weights.

**Table 5:** IG2 values for yeast protein interactions

P_name 1	P_name 2	PID_1	PID_2	t1	t2	t3	t4	t5	IG2
AAC1	YHR005C-A	1	1913	0	0	0	2	2	26.94071
ANC1	SNF5	1	3246	0	0	0	3	0	26.90991
ANC1	TAF25	1	3517	0	0	164	5	3	-121.486
ABP1	ACT1	19	33	2	0	4	10	6	26.97287
ABP1	RVS167	19	2980	1	1	2	13	0	23.08996
ABP1	SRV2	19	3384	1	1	2	12	0	24.42532
YER045C	PSE1	22	2483	0	0	2	3	1	24.44631
ACC1	DMC1	24	785	0	0	0	20	3	25.10544
ACC1	SNP1	24	3258	0	0	0	10	13	26.56783
ACE2	YNL157W	25	5838	0	0	0	0	1	26.8268
ACS2	SNP1	32	3258	0	0	0	7	12	26.97778
ACT1	AIP1	33	84	0	0	8	10	6	26.88486
ACT1	BEM1	33	304	0	2	20	14	7	26.97287
ACT1	BNI1	33	333	2	2	4	14	7	19.55493
ACT1	BUD6	33	370	1	1	10	22	7	7.772698
ACT1	CAP2	33	407	0	0	8	10	6	22.16398
ACT1	COF1	33	568	0	0	8	10	5	17.03328
ACT1	FUS1	33	1065	0	0	8	10	6	19.55493
ACT1	GLK1	33	1164	0	0	8	7	9	19.55002
ACT1	IQG1	33	1470	0	2	8	13	6	19.55493
ACT1	LAS17	33	1583	0	0	8	9	7	19.63262
ACT1	MYO4	33	1983	0	0	8	10	5	18.64504
ACT1	OYE2	33	2174	0	0	8	10	5	19.58083
ACT1	PFY1	33	2295	1	1	6	11	6	19.55002
ACT1	RPP2B	33	2843	0	0	8	9	7	19.55002

**Table 6:** The protein interactions and their suggested weights

Protein A name	Protein B name	Protein A-ID	Protein B-ID	W0	W1	W2	W3	W4	W5
AAC1	YHR005C-A	1	1913	1	0.5	1	0.5	0.66	0.5
ANC1	SNF5	1	3246	1	1	1	0.5	0.83	1
ANC1	TAF25	1	3517	1	0.5	0.5	1	0.66	1
ABP1	ACT1	19	33	1	1	0.5	0.5	0.66	1
ABP1	RVS167	19	2980	1	1	1	0.5	0.83	1
ABP1	SRV2	19	3384	1	1	1	0.5	0.83	1
YER045C	PSE1	22	2483	1	0.5	1	0.5	0.66	0.5
ACC1	DMC1	24	785	1	0.5	0.5	0.5	0.5	0.5
ACC1	SNP1	24	3258	1	0.5	0.5	0.5	0.5	0.5
ACE2	YNL157W	25	5838	1	0.5	1	0.5	0.66	0.5
ACS2	SNP1	32	3258	1	0.5	0.5	0.5	0.5	0.5
ACT1	AIP1	33	84	1	0.5	0.5	0.5	0.5	1
ACT1	BEM1	33	304	1	1	0.5	1	0.83	1
ACT1	BNI1	33	333	1	0.5	0.5	0.5	0.5	0.5
ACT1	BUD6	33	370	1	0.5	0.5	1	0.66	1
ACT1	CAP2	33	407	1	1	0.5	0.5	0.66	1
ACT1	COF1	33	568	1	1	0.5	0.5	0.66	1
ACT1	FUS1	33	1065	1	0.5	0.5	0.5	0.5	1
ACT1	GLK1	33	1164	1	1	0.5	0.5	0.66	1
ACT1	IQG1	33	1470	1	1	0.5	1	0.83	1

### Integration process

After collecting the levels for each protein and the five different weights, the neighbour counting method (frequencies of interaction partners having certain functions of interest) was implemented to predict the functions of unknown proteins. The new weights and weightless (edges with equal weights (W0)) algorithms were compared for proteins having up to five interactions. This demonstrated that for most selected new weights at a specific specificity (SP), the sensitivity (SN) was higher than obtained using W0. As demonstrated in Figures 1, 2 and 3, the sensitivity and the specificity of the three function categories of yeast (Biochemical, Cell location and Cellular role) are indicated respectively. Equations 1 and 2 present the basic laws of SN and SP.

The sensitivity (SN) and specificity (SP) can be defined as:(1)(2)

Where *n_i _*is the number of *observed *functions for protein *P_i_*

*m_i _*is the number of *predicted *functions for protein *P_i_*,

and *k_i _*are the overlaps between them.

## Competing interests

The authors declare that they have no competing interests.

## Authors' contributions

KS collected the biological data, participated data implementation and drafted the manuscript. NS participated in the statistical analysis. YK conceived of the study, and participated in its design and coordination. All authors read and approved the final manuscript.
